# Micro-Structural and Biomechanical Evaluation of Bioresorbable and Conventional Bone Cements for Augmentation of the Proximal Femoral Nail

**DOI:** 10.3390/jcm12237202

**Published:** 2023-11-21

**Authors:** Christoph Linhart, Manuel Kistler, Maximilian Saller, Axel Greiner, Christopher Lampert, Matthias Kassube, Christopher A. Becker, Wolfgang Böcker, Christian Ehrnthaller

**Affiliations:** Department of Orthopaedics and Trauma Surgery, Musculoskeletal University Center Munich (MUM), University Hospital, LMU Munich, Marchioninistr. 15, 81377 Munich, Germany; manuel.kistler@med.uni-muenchen.de (M.K.); maximilian.saller@med.uni-muenchen.de (M.S.); axel.greiner@med.uni-muenchen.de (A.G.); christopher.lampert@med.uni-muenchen.de (C.L.); matthias_kassube@hotmail.de (M.K.); christopher.becker@med.uni-muenchen.de (C.A.B.); wolfgang.boecker@med.uni-muenchen.de (W.B.); christian.ehrnthaller@med.uni-muenchen.de (C.E.)

**Keywords:** bone cement, PMMA, micro-computed tomography, biomechanics, proximal femur fracture, osteoporosis, cement augmentation, subtrochanteric fracture

## Abstract

Osteoporotic proximal femur fractures are on the rise due to demographic change. The most dominant surgical treatment option for per/subtrochanteric fractures is cephalomedullary nailing. As it has been shown to increase primary stability, cement augmentation has become increasingly popular in the treatment of osteoporotic per/subtrochanteric femur fractures. The ultimate goal is to achieve stable osteosynthesis, allowing for rapid full weight-bearing to reduce possible postoperative complications. In recent years, bioresorbable bone cements have been developed and are now mainly used to fill bone voids. The aim of this study was to evaluate the biomechanical stability as well as the micro-structural behaviour of bioresorbable bone cements compared to conventional polymethylmethacrylate (PMMA)-cements in a subtrochanteric femur fracture model. Biomechanical as well as micro-computed tomography morphology analysis revealed no significant differences in both bone cements, as they showed equal mechanical stability and tight interdigitation into the spongious bone of the femoral head. Given the positive risk/benefit ratio for bioresorbable bone cements, their utilisation should be evaluated in future clinical studies, making them a promising alternative to PMMA-bone cements.

## 1. Introduction

Due to the change in demographics, the incidence of proximal femur fractures will increase over the next few years and represent a major health care issue [1]. Elderly patients often present with multiple comorbidities, leading to a significant increase in perioperative and postoperative complications. As a result, proximal femur fractures are associated with a high one-year mortality rate of up to 30% [2]. The treatment goal of proximal femur fractures must therefore ensure the preservation of function and independence in orthogeriatric patients [3]. Among proximal femur fractures, the per/subtrochanteric fracture is most common, followed by femoral neck fractures [4]. Whereas femoral neck fractures are mostly treated with hip joint arthroplasty, joint-preserving osteosynthesis is the predominant surgical treatment for per/subtrochanteric fractures. Osteosynthesis in pertrochanteric fractures is either performed using a dynamic hip screw (DHS, DePuySynthes^®^ Inc., Oberdorf, Switzerland) or a proximal femur nail with a hip component. The current evidence does not promote one solution, whereas a trend towards the use of intramedullary implants is observed [5,6,7]. This study focuses on reverse-oblique subtrochanteric fractures, especially AO (Arbeitsgemeinschaft für Osteosynthesefragen) 31-A3. Being the most unstable fracture type among all proximal femur fractures, predominantly intramedullary implants are used.

As already mentioned, the most common treatment option for per/subtrochanteric femur fractures nowadays is nailing of the proximal femur. High initial stability due to intramedullary load distribution together with short duration of the surgical procedure have led to an increase in the use of the dynamic hip screw. Additionally, these fractures of the femur are predominantly associated with osteoporosis. Despite the reduced biomechanical bone stability and an increased perioperative risk in prevalent osteoporosis, a surgical procedure that allows rapid full weight-bearing mobilisation must be achieved [1], as a significant increase in mortality was observed when patients were treated with weight-bearing restrictions [8]. Cement augmentation with polymethylmethacrylate (PMMA) of proximal femoral nailing (PFNA) for the treatment of pertrochanteric femoral fractures is the most commonly used and standardised method to deal with that problem [3], as it has been shown to increase primary stability. The additional augmentation increases the contact area of the implant-bone interface. Biomechanical investigations were already able to demonstrate the higher stability of the cement-augmented PFNA for the treatment of osteoporosis-associated fractures when compared to the non-cemented control [9,10]. Prospective studies showed good functional results with no evidence of cement-associated complications for cement augmentation with PMMA of the PFNA in orthogeriatric patients [11,12,13]. Although augmentation of PFNA did not affect the walking ability in the early postoperative period, it led to significantly fewer implant failure- or migration-caused reoperations. This implicates a higher stability of the osteosynthesis construct, allowing a fast full weight-bearing mobilisation [13].

However, PMMA also faces some points of criticism, such as thermal injury to surrounding tissues [14], possible intraarticular leakage, and its failing ability for resorption and transformation into bone. Therefore, various surgeons deny cement augmentation, even though it would be possible [15].

To avoid these negative side effects, bioresorbable liquid bone substitutes with similar physical properties to PMMA and high mechanical strength due to curing processes were developed during the last few years. Bioresorbable bone cement consists of both α-CaS hemihydrates and hydroxyapatite (HA) [16]. The calcium sulphate will be resorbed and replaced by in-growing bone, whereas the hydroxyapatite component persists and functions as a matrix. Thereby, it combines the advantage of immediate stability with the osteoconductive characteristics of a bone substitute material [17,18].

In its first intention, bioresorbable bone cement was used as an alternative for autologous bone grafting in traumatic injuries with bone loss [19]. Moreover, several studies investigated its use for the filling of bone cysts [20], as well as balloon kyphoplasty, and verified the safety of the procedure [21]. In addition, clinical studies showed that bioresorbable bone cements could be used instead of PMMA in the treatment of osteoporotic and traumatic vertebral fractures [22]. However, so far, bioresorbable bone cements have never been tested as an alternative for augmentation of osteosynthesis implants, although the advantages of failing exothermal reactions and biodegradable properties are obvious. It has to be taken into account that bioresorbable bone cements represent a very heterogeneous group of substances that are commercially available from various manufacturers with different characteristics regarding application capability, strength, and resorption.

The aim of this study was to evaluate bioresorbable cement for the augmentation of PFNA in proximal femur fractures. We hypothesised comparable biomechanical as well as physical properties that will allow a safe use together with a proximal femur nail. Therefore, we compared cement augmentation with bioresorbable cement to cement augmentation with conventional, commercially used PMMA. In addition, biomechanical studies examining the primary stability of each type of cement, micro-structural properties (distance between trabecular structures and cement before and after loading) were analysed by micro-computed tomography (µCT). Since its introduction in the 1980s, µCT has become an essential tool in musculoskeletal research and plays a pivotal role in the evaluation of bone microarchitecture in small animal models ex vivo [23]. In our study, we were the first to use long human tubular bones to visualise the trabecular-cement interface in particular.

## 2. Materials and Methods

After ethical approval from the Human Research Ethics Committee of the University of Munich, LMU, Germany (No. 8-030), 10 fresh frozen human femora of female donors >75 years were commercially obtained from Science Care (Phoenix, AZ, USA). The 10 human femora were split up into two groups, each consisting of five specimens. The femora were frozen at −24 °C and thawed at room temperature for 24 h before experimental testing. To increase comparability and reduce bias, only pairs (both femora) from each donor were used. A short PFNA (L = 240 mm, D = 10 mm) (PFNA, DePuySynthes^®^ Inc., West Chester, PA, USA) was implanted in each of these human specimens according to the official manufacturer’s instructions. For the purpose of minimally invasive implantation and standardisation, the original manufacturer’s instrumentation was used. The fracture was set standardised according to an AO 31-A3 fracture that is 50° oblique in terms of a “reverse-oblique” fracture. After successful implantation of the PFNA, the fracture was marked on the intact bone. The angle was determined by the shaft axis. The osteotomy was carefully performed with a hand saw without damaging the nail.

Afterwards, all specimens were tapered at the distal end and embedded under a 6° valgus position in metal pots using a polyurethane resin (RenCast^®^ FC 52/53 Isocyanat; FC 53 Polyol, Huntsman Corporation, The Woodlands, TX, USA). Five specimens were augmented using classical PMMA-cement (Traumacem; DePuy Synthes^®^ Inc., West Chester, PA, USA) and five using bioresorbable bone cement (Cerament; BoneSupport, Lund, Sweden). Augmentation for both cements was performed with an application instrument for the PMMA-cement under fluoroscopic control until a uniform distribution using 3–4 mL of cement in the femoral head was achieved. The function of the iliotibial tract was simulated with a 3 mm steel cable that becomes continuously stretched with increasing load. To measure the force characteristic of the simulated tractus iliotibialis during force application of the bone, a 5 kN load cell (8417–6005, Burster Praezisionsmesstechnik GmbH and Co. KG, Gernsbach, Germany) was implemented in the wire rope. A program was created in LabVIEW 2014 (Version 14.0.1, National Instruments, Austin, TX, USA) to calibrate the sensor and collect the force data. Sensor calibration was performed using the universal testing machine. Specified tensile and compressive forces in the range from −200 N (compressive force) to +1500 N (tensile force) were applied to the force sensor in 100 N increments. A 3D ultrasound system (Zebris CMS 20, Zebris Medical GmbH, Isny, Germany) was used to track the scope of 3D movement of the bone segments to measure the displacement of the fracture gap in three dimensions in millimeters. Depending on the application, the system has a resolution of 1/10 mm − 1/100 mm with a measurement error of 0.25%. These ultrasound markers were screwed in place by an additively manufactured holder or, in some special cases, directly to the bone with hot glue. All tests were performed on an electrodynamic universal testing machine (ElectroPulsTM E10000, Instron, Norwood, IL, USA). The simulated tractus iliotibialis was slightly preloaded before loading until an effective joint force of 50 N was applied on the testing machine. Afterwards, biomechanical testing of the instrumented specimens was performed under displacement-controlled force application at 10 mm/min up to effective joint loads of 200 N and 400 N without creating a failure. All load procedures were repeated three times to avoid possible settling of the implant. Biomechanical stability was assessed by measuring the amount of fracture displacement and axial stiffness of the bone construct (N/mm) of the instrumented specimen. Stiffness was defined as the resistance of the bone structure against elastic deformation in response to an applied load without considering the geometry of the cross section. Additionally, force distribution of the iliotibial tract (N) during force application of the bones was measured as a secondary parameter for dislocation due to the expected tension release following fragment dislocation. For the µCT, due to the size of the femora and the need to rotate the specimens, a special bone holder was made using a 3D printer (Ultimaker 2+ Extended, Ultimaker B.V., Utrecht, The Netherlands). This was achieved by creating a computer-aided 3D model (Catia V5, Dassault Systemes, Velizy-Villacoublay, France) based on the bone size.

To obtain high-resolution images of the trabecular-material interface, the femora were imaged with a custom-build research µCT (CT-Alpha, Procon X-Ray, Sarstedt, Germany), equipped with an nanofocus X-ray source (XWT-225-TCHE, X-Ray-Worx, Germany) set at 200 kV/240 mA (with a 1 mm aluminium filter) and a 75 µm pixel size CMOS flat panel detector (Dexela 2923, PerkinElmer, Santa Clara CA, USA) at a 2 × 2 binning. 1440 images with an exposure time of 250 ms and a two-time averaging were recorded over a full 360° rotation. All scans were performed both before and after biomechanical testing and cement application. The distance between trabecular structures and cement was measured in each of the 10 regions of interest (ROI). The acquired raw data was reconstructed with X-Aid (Mitos, Germany) after correction of geometrical errors and beam hardening.

After the computed tomography images were taken, the Dragonfly software (Object Research Systems (ORS) Inc, Montreal, QC, Canada, 2020) was used to analyse the images.

A region of interest was created for each plane and coloured through all the images of that plane to measure the progression between cement and trabeculae within a specimen, always at the same points.

**Figure 1:**
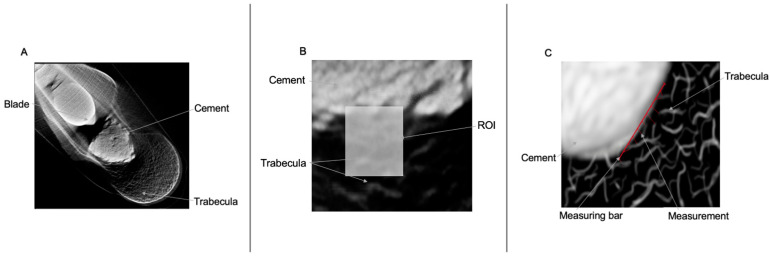
(**A**): Overview in micro-computed tomography of the cement-trabecular interphase of the proximal femur; (**B**): detail of the ROI; (**C**): schematic drawing of the ROI and the points of evaluation of the cement-trabecular interphase.

For each of the three planes (axial, sagittal, longitudinal), a line of 9 mm length was then placed at the ROI. 10 measurements were taken, starting at 0 mm and with 1 mm between each measurement (Figure 1).

For each measurement, the distance between the cement and the nearest trabeculae was measured. Once 10 measurements were taken, the measurement procedure was performed again, five frames forward from the last one.

When cement was no longer visible in the ROI of a plane, the measurement was stopped and moved to the next plane.

### Statistical Analysis

For data collection and analysis, the software SPSS (IBM Corp. Released 2021) was used. IBM SPSS Statistics for Macintosh, Version 28.0. Armon, NY, USA: IBM Corp) and Excel (Microsoft, released 2021, Microsoft Excel for Macintosh, Version 16.50) were used. All charts were created with Prism 9 (GraphPad Software, Inc., La Jolla, CA, USA).

To evaluate the data for homogeneity of variances, the Levene test was used. Normal distributed data was analysed using the *t*-test for paired or unpaired samples. For non-normally distributed data, the Wilcoxon test (paired) or the Mann-Whitney-U test (unpaired) were used. Differences with a *p*-value < 0.05 were defined as significantly different.

## 3. Results

### 3.1. Biomechanics

All results of the biomechanical testing (Table 1) were normally distributed and showed no significant difference between the two types of cement. In detail, the *p*-value of the dislocation in the fracture gap between the cements resulted at 200 N in a slightly reduced displacement of 9.8% for the bioresorbable cement group (*p* = 0.65) and at 400 N in an almost evenly distributed displacement (1.7% less in the bioresorbable cement group; *p* = 0.89). The axial stiffness of the bone construct revealed a non-significant (*p* = 0.33) reduction in the PMMA-cement group of 25.4% at 200 N and a 2.9% reduction at 400 N (*p* = 0.67). Additionally, the force of the iliotibial tract indicated no significant differences at 200 N (*p* = 0.53) and 400 N (*p* = 0.10).

#### 3.1.1. PMMA-Cement

**Figure 2:**
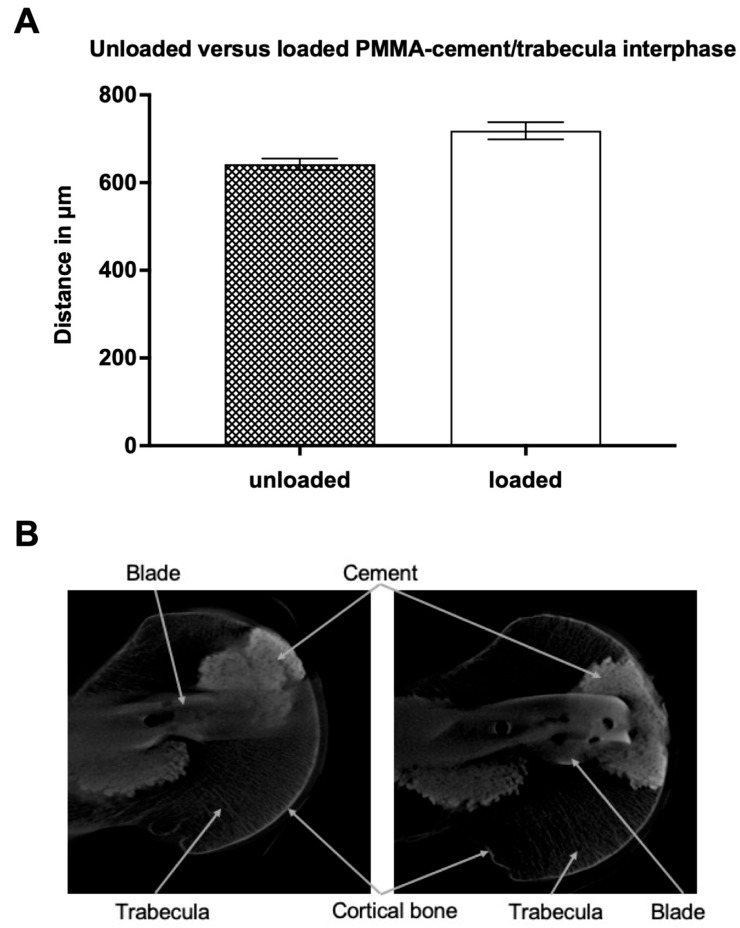
(**A**): Average distance (in micrometers) between unloaded versus loaded PMMA-cement and the closest trabecular structure. Data presented as mean ± SEM (standard error of the mean); (**B**): Unloaded (**left**) vs. loaded (**right**) PMMA-cement/trabecula interphase picture of the micro-CT. The specimen was treated with a proximal femur nail and augmented with PMMA-cement before a CT scan was taken.

The average distance between the cement and the trabeculae in the PMMA-cement group before conducting the biomechanical test was 642 ± 13 (SEM) μm. After loading, the mean value in the PMMA-cement group increased up to 718 ± 20 (SEM) μm (Figure 2). Therefore, the distance between cement and trabeculae has increased by 12%. The statistical analysis showed no significant difference (*p* = 0.87).

#### 3.1.2. Bioresorbable-Cement

**Figure 3:**
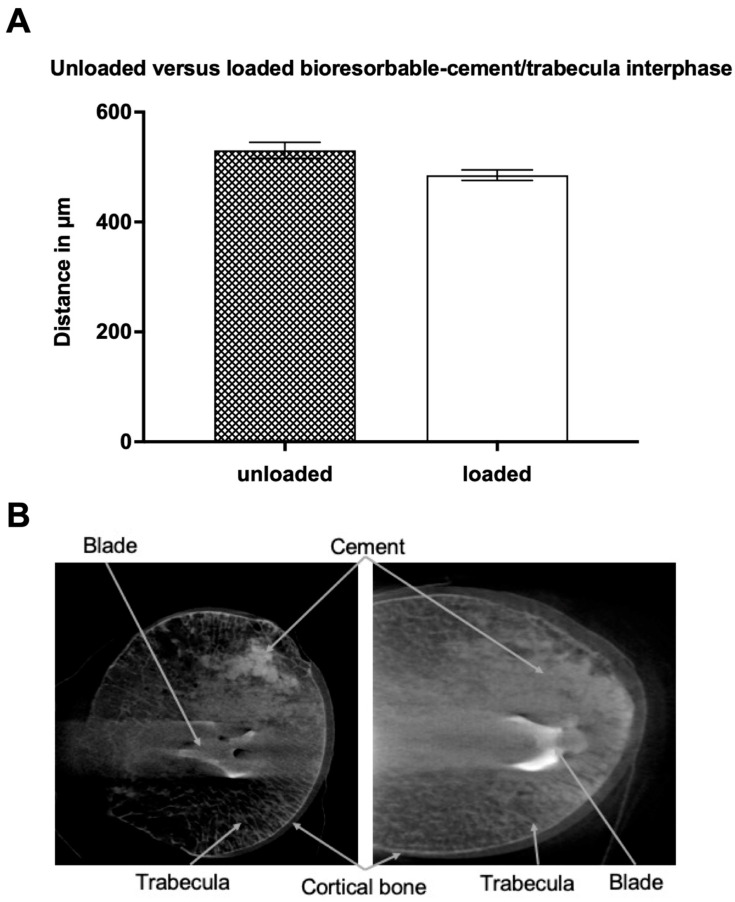
(**A**): Average distance (in micrometers) between unloaded versus loaded bioresorbable cement and the closest trabecula structure. Data presented as mean ± SEM (standard error of the mean); (**B**): Unloaded (**left**) vs. loaded (**right**) bioresorbable-cement/trabecula interphase picture of the 5 micro-CT. The specimen was treated with a proximal femur nail and augmented with bioresorbable cement before a CT scan was taken.

In the bioresorbable cement group, the average distance between cement and trabeculae before the biomechanical test was 530 ± 15 (SEM) μm. After biomechanical loading, the average distance of the bioresorbable-cement group decreased to 485 ± 10 (SEM) μm (Figure 3). This difference of 8.5% was significantly different (*p* = 0.03).

### 3.2. Micro-Computed Tomography

#### 3.2.1. Unloaded PMMA-Cement vs. Bioresorbable-Cement

**Figure 4:**
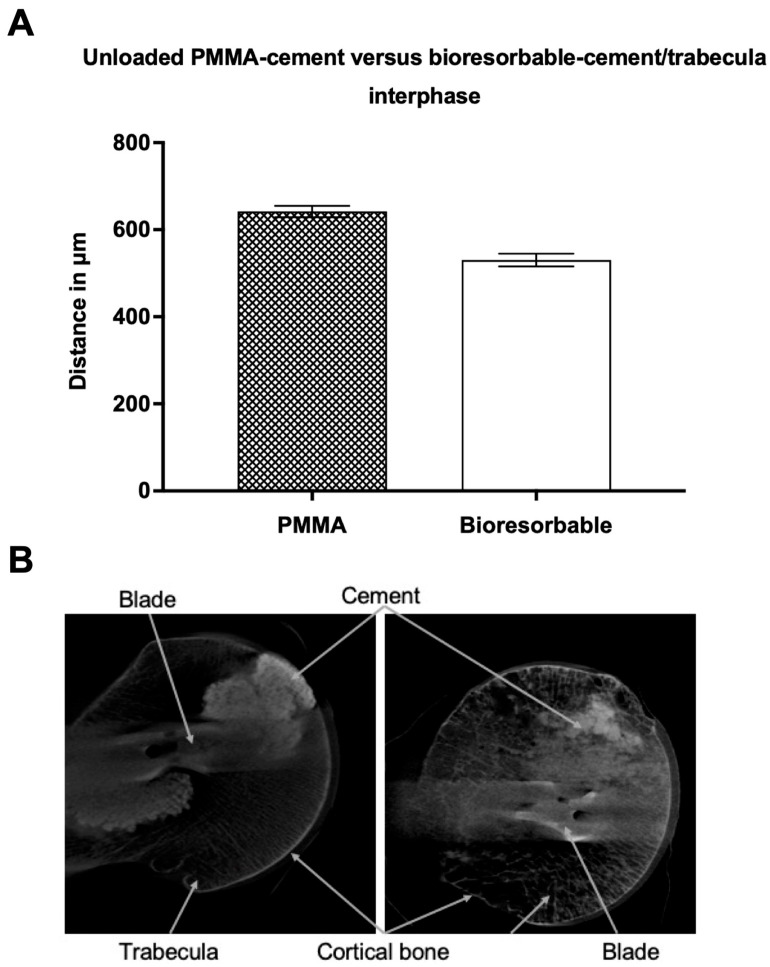
(**A**): Average distance (in micrometers) between unloaded PMMA-cement versus unloaded bioresorbable-cement and the closest trabecular structure. Data presented as mean ± SEM (standard error of the mean); (**B**): Unloaded PMMA (**left**) vs. unloaded (**right**) bioresorbable-cement/trabecula interphase picture of the micro-CT. The specimen was treated with a proximal femur nail and augmented with PMMA- or bioresorbable-cement before a CT scan was taken.

While comparing the two different types of cement before the biomechanical experiment, more profound differences were evaluated. The average cement-trabecular distance in the unloaded PMMA-cement group was 642 ± 13 (SEM) μm when compared to 530 ± 15 (SEM) μm in the group with bioresorbable cement (Figure 4). Statistical analysis showed a significant difference (*p* ≤ 0.0001) between the two groups. It turns out that the cement-trabeculae interphase before loading has a 17% tighter structure than in the PMMA group before loading (Figure 4). 

#### 3.2.2. Loaded PMMA-Cement vs. Bioresorbable-Cement

**Figure 5:**
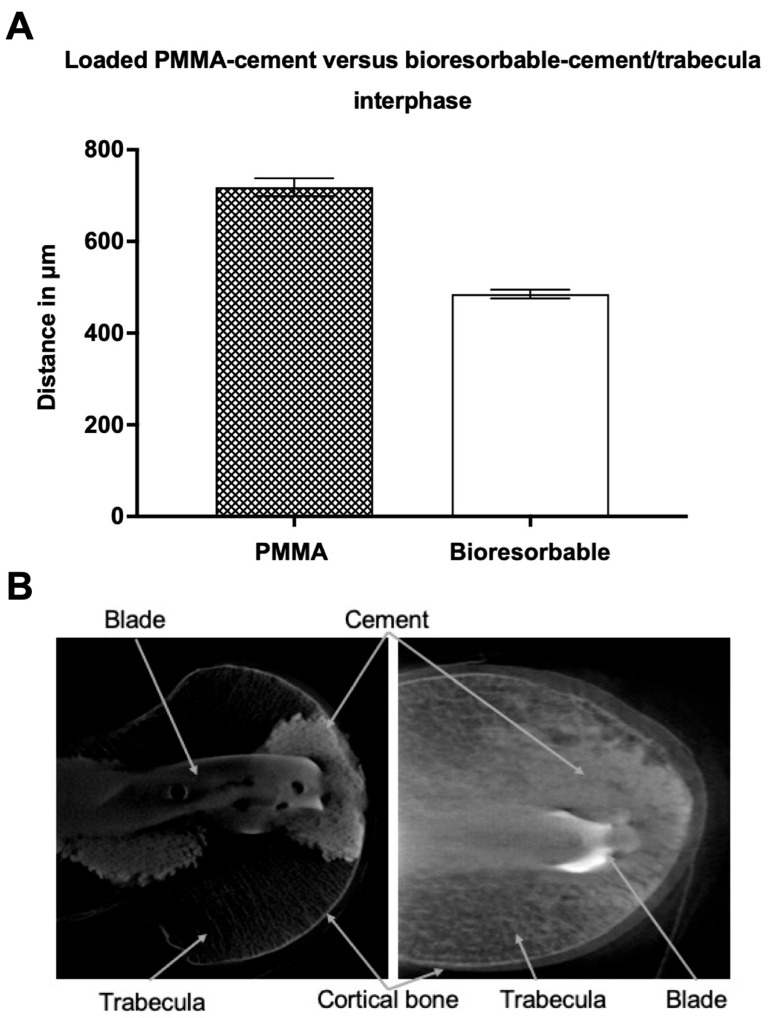
(**A**): Average distance (in micrometers) between loaded PMMA-cement versus loaded bioresorbable-cement and the closest trabecular structure. Data presented as mean ± SEM (standard error of the mean); (**B**): Loaded PMMA (**left**) vs. loaded (**right**) bioresorbable-cement/trabecula interphase picture of the micro-CT. The specimen was treated with a proximal femur nail and augmented with PMMA- or bioresorbable-cement before a CT scan was taken.

Biomechanical loading of the specimens was able to confirm the trend towards higher indentation in the bioresorbable-cement group as seen before loading. While the average cement-trabecular distance in the PMMA-cement group was 718 ± 20 (SEM) μm, the bioresorbable-cement group showed values of 485 ± 10 (SEM) μm (Figure 5). There was a significant difference (*p* ≤ 0.0001) between the two groups, which was reflected in a 32.5% tighter cement-trabeculae interphase in the structure of the bioresorbable cement.

## 4. Discussion

In this study, the micro-structural and biomechanical properties of PMMA as well as bioresorbable bone cements used for augmentation of the femoral head were compared for the first time in a subtrochanteric fracture model.

We were able to demonstrate that the use of bioresorbable bone cement in an experimental unstable subtrochanteric femur fracture model resulted in a tight interaction of the injected cement with the surrounding trabecular bone with biomechanical properties similar to classic PMMA-bone cement after the conduction of a classical loading test.

During the last few years, the augmentation of implants used for osteosynthesis has become increasingly popular [13]. While this technique was used primarily in proximal femur fractures [24], its usage has been expanded to include other osteoporotic fractures such as proximal humerus, insufficiency fractures of the sacrum, and vertebral fractures [25,26]. Possible advantages include the increase of primary fixation strength [10] to prevent secondary dislocation and implant failure and, therefore, ultimately achieve mobilisation and weight-bearing as soon as possible. Various biomechanical studies were already able to demonstrate an increase in primary fixation strength compared to non-augmented proximal femoral nails [10]. A randomised clinical trial was able to demonstrate the safety of the augmentation of the femoral head with PMMA with comparable clinical outcomes and increased cost-effectiveness due to reduced implant failure rates [13,27].

Currently, PMMA bone cement is the only substance approved for augmentation of osteosynthesis implants. Special compositions from various manufacturers with favourable injection characteristics for their distinct type and application location were developed during the last few years to prevent possible risks during application. Although, in clinical studies, no augmentation-related negative side-effects have been reported so far [2,11,12] possible risks such as bone/cartilage necrosis due to the exothermic reaction while hardening as well as leakage (e.g., intraarticular in fractures close to joints), and intravasal (e.g., spinal vertebrae during vertebral kyphoplasty) are often mentioned and still raise concerns among orthopaedic surgeons. Only recently, a study focussing specifically on postoperative complications after augmentation of the femoral head in femur fractures was published. In addition, a small drop in blood pressure indicating a small systemic effect of the cement application, no significant alterations regarding postoperative complications such as infection, embolism, heart-attack, or mortality were seen [28].

With the intention of avoiding possible negative side-effects of PMMA and a hypothetical benefit of a biodegradable substance, the development of bioresorbable bone cements has been fostered throughout the last few years. These bone cements were initially developed to fill up bone voids as an alternative to autologous bone, which is limited in the amount that can be harvested [19].

Being a heterogeneous group of substances, bioresorbable bone cements have become increasingly sophisticated, being fluid in the application phase with uniform distribution and then becoming increasingly biomechanically stable after hardening [29]. In addition, being used as a bone void filler in the beginning, bioresorbable bone cements have become popular in vertebral compression fractures [21], osteomyelitis [30], and bone cysts [20]. The utilisation of bioresorbable bone cements in fractures with the need for bone grafting as an alternative to autologous bone has only recently been investigated and found to be similar in terms of outcome but superior in terms of blood loss and postoperative pain in comparison to autologous iliac crest bone grafting [19].

Just recently, a similar study to ours investigated the utilisation of a custom-made magnesium-based bioresorbable cement for its use in augmentation of the femoral head. In contrast to our study, the biomechanical stability of bioresorbable cement was inferior in comparison to PMMA. A possible explanation could be seen in the different characteristics of the used cement types [31]. In contrast to the commercially available CaS-based bone cement used in our study, implant augmentation was performed using an experimental custom-made magnesium-based cement. It is noteworthy, that the experimental setup differed greatly from ours, limiting comparability. Instead of testing the proximal femur during an experimental setup with axial load and additional simulation of the tractus iliotibialis, only a rotational test of the augmented femoral head implant was performed in the latter study. Therefore, it is tempting to speculate that a commercially available CaS-based bioresorbable bone cement seems to be more favourable for the use of implant augmentation.

Generally speaking, the advantageous characteristic properties of bioresorbable bone cement in comparison to classical PMMA-cement are obvious. They do not result in high temperatures during hardening with possible bone/cartilage damage, provide biomechanical stability, and are replaced by autologous bone over time [32].

As shown in this study, the application of bioresorbable bone cement through commonly used applicators for PMMA-cement was performed without any limitations and was safe. Microstructural evaluation showed a close interaction of the bioresorbable bone cement with the autologous trabecular bone structures. Objective evaluation showed an even closer indentation of bioresorbable cement in comparison to classical PMMA-cement by 21%.

Not only was the application of the bioresorbable cement safe, but it also led to a good distribution in the femoral head. Biomechanical evaluation with a non-destructive load test overall showed similar primary stability of the bioresorbable cement compared to PMMA-cement. At a closer look, the bioresorbable cement even showed a non-significantly higher stability of 25% at 200 N, which decreased to 3% at 400 N, possibly due to a settling effect.

### Limitations of the Study

The major limitation of our study is the relatively small sample size. The true intergroup differences may have been more profound with a larger sample size.

Additional limitations arise from the chosen study design’s cross-sectional character and the individual variability of donor specimens, with unknown underlying comorbidities possibly affecting the results.

The utilisation of human fresh frozen specimens may influence the distribution and reactivity of the injected bone cements. However, besides clinical application in humans, no superior study design exists for performing a feasibility study.

## 5. Conclusions

The reduced distance between trabecular bone and cement in the group of bioresorbable cement is an indication of better interlocking of the cement in the surrounding bone, which can be explained by the more fluid consistency of the bioresorbable cement. The fact that after loading, the cement/bone distance decreases in the bioresorbable-cement group but increases in the PMMA-group could be an expression of the different fatigue strength of PMMA-cement compared to the bioresorbable-cement. Whereas it can be suspected that PMMA-cement will not change its structure during loading, bioresorbable-cement might show some settling during loading, possibly explaining the closer indentation after loading. Although settling could be suspected with a decrease in biomechanical strength, no significant differences between PMMA-cement and bioresorbable-cement were observed in regard to dislocation of the fracture gap, axial stiffness, and the force curve of the tractus iliotibialis. Therefore, bioresorbable bone cement seems to inherit all the beneficial properties to be suitable for augmentation of osteosynthesis implants, and their use should be evaluated in a clinical study as the next step.

## Figures and Tables

**Table 1:** Results of the biomechanical tests between two bone cements at different test loads.

Test load	200 N		400 N	
Cement	PMMA	Bio-Cement	PMMA	Bio-Cement
Fracture displacement in mm	1.13 ± 0.39	1.02 ± 0.32	1.22 ± 0.31	1.20 ± 0.26
Axial bone stiffness in N/mm	31.13 ± 12.75	41.73 ± 16.66	30.16 ± 2.34	31.08 ± 3.60
Iliotibial tract force in N	345.00 ± 33.76	334.80 ± 8.03	740.80 ± 53.80	695.00 ± 13.45

## Data Availability

The data presented in this study are available on request from the corresponding author. The data are not publicly available due to privacy reasons.
